# Chronic Colitis

**Published:** 1907-12-14

**Authors:** 


### Diseases of Children.

### CHRONIC COLITIS.

After the period of infancy the occurrence of chronic colitis in children is comparatively infrequent. This is fortunate, for in many instances the affection is one particularly troublesome to get rid of. Possibly some of the cases of chronic mucous colitis in adults are the sequel of neglected or ill-managed attacks in early life.

The actual causation of the chronic inflammation is often a matter of doubt. As a general rule an inquiry into the previous history will reveal either neglected constipation or an acute attack of entero-colitis. Chronic mild constipation produces it through the constant irritation of the mucous membrane by scybala. Acute entero-colitis produces an acute congestion of the mucosa, which for some reason or other does not entirely get well.

### Clinical Types.

There are various types of the affection. The most frequent and the mildest form is a simple catarrhal inflammation in which the mucous membrane is swollen, red, and thickened. Sometimes it is associated with superficial abrasion or ulceration of the mucous membrane, leading to outbreaks of hæmorrhage. Bleeding may also occur from mere congestion. In another type, rarely seen after the period of infancy, the colon is more or less studded with small follicular ulcers. A more serious state of ulceration is sometimes present, due either to the extension in surface and depth of the superficial destruction of tissue or to the confluence of numerous small follicular ulcers. Finally, there is a rare variety, the true membranous colitis, in which the surface of the colon is covered, in patches or universally, by adherent membrane which may be evacuated in the form of tubular casts.

These different varieties are named (1) catarrhal or mucous, (2) follicular, (3) ulcerative, (4) membranous.

### Symptoms and Signs.

One troublesome feature in the cases of catarrhal colitis is that the symptoms are so slight at first that it is frequently overlooked. It is quite common for the child to be brought because, for " some months " or " for a year," and possibly longer, mucus has been noticed in the stools. The mucus resembles slime, jelly-like masses, or white of egg. It is generally on the surface of the fæces and not intimately mixed therewith, and may be occasionally passed alone in quantities as great as a wineglassful at a time. Yet the subjective symptoms are slight or absent. The child may appear perfectly well. Frequently there is a history of languor, disinclination to play, dark rings under the eyes, and perhaps a variable appetite. Often, however, the appetite remains excellent throughout. In the more serious cases there is the evidence of blood in the stools, fairly bright in colour, variable in amount and in frequency of evacuation. Some children will, under careful treatment, pass apparently normal stools for two or three weeks at a time, and then, for no apparent reason, fresh hæmorrhage occurs. It is particularly liable to be induced by increased diet, indigestible food, and exertion. The stools in the hæmorrhagic cases are often loose, frequent, and very offensive, more especially if there is marked ulceration. Probably when they are formed and infrequent there is no appreciable amount of ulceration, merely an intense congestion of the mucosa.

Physical examination reveals little, except, perhaps, in the severe ulcerative type, in which tenderness may be found along the course of the colon; not necessarily throughout, for it may be limited to one particular portion, corresponding with the area of greatest mischief. Thus I have seen children in whom the tenderness was marked over the transverse colon, descending colon, and sigmoid region. In every case a rectal examination should be made to exclude the possibility of polypus, papilloma, sarcoma, angeioma, and local ulceration or foreign body in the rectum. By using the sigmoidoscope it may be possible to see what the actual state of the mucosa is, and even to locate ulceration if it is low down.

### Treatment.

The treatment of the disease is difficult, because, from the nature of the case, the inflamed surface is exposed to the irritation of the products of digestion, and still more because parents will not always realise that the disease is much more serious than it appears, is very difficult to cure, and necessitates prolonged care, sometimes for months. A guarded prognosis should be given. In the long run these patients almost invariably recover, but they are liable to relapse, and it is especially hard to be sure that any particular child is really cured.

Mild cases will recover on simple dietetic treatment, warmth, bismuth, and attention to the bowels. Avoid all foods leaving an indigestible residue, notably vegetables. Give frequent doses of bismuth and a saline purge, such an Apenta water, every morning. In more severe attacks it is necessary to keep the child in bed, on a diet of peptonised milk with the addition of lactose and one or two eggs. Iodoform pills or creosote mixtures by mouth are often useful if the stools are offensive. Mild purgatives or enemata should be given for constipation; large doses of salicylate of bismuth and pulv. cret. aromat. for profuse diarrhœa. The use of curdled milk or the administration of lactic-acid bacilli is sometimes of value in disinfecting the intestinal tract, the proteolytic organisms being apparently destroyed or crowded out by these bacilli. Finally, we can fall back on irrigation of the colon by mild antiseptics or astringents. In the ulcerative cases nitrate of silver may prove of very great service. Supposing all else fails and the child is getting into an apparently hopeless state, the question of irrigation from above, after appendicostomy, must be considered.

